# Soil applied glycine betaine with *Arbuscular mycorrhizal fungi* reduces chromium uptake and ameliorates chromium toxicity by suppressing the oxidative stress in three genetically different Sorghum (*Sorghum bicolor* L.) cultivars

**DOI:** 10.1186/s12870-021-03113-3

**Published:** 2021-07-14

**Authors:** Praveen Kumar

**Affiliations:** Department of Biochemistry, College of Basic Sciences and Humanities, Chaudhary Charan Singh Haryana Agricultural University, Hisar, Haryana 125004 India

**Keywords:** Amelioration, Antioxidants, Chromium, Glycine betaine, AMF, Sorghum, Toxicity, Quenching

## Abstract

**Background:**

Chromium is the most toxic pollutant that negatively affects a plant’s metabolic activities and yield. It reduces plant growth by influencing the antioxidant defence system’s activities. In the present study, a completely randomized block design experiment with three plants/pot in three replication was conducted on three varieties of sorghum *viz.* SSG 59–3, HJ 513 (multi-cut) and HJ 541 (single-cut) for amelioration of chromium toxicity (2 & 4 ppm) by exogenous application of GB (50 & 100 mM) with and without AMF in soil. The ameliorative effects were tested at two growth stages *viz.* vegetative (35 DAS) and grain filling (95 DAS), in terms of Cr uptake, grain yield, antioxidative defence system parameters (*viz.* enzymes – SOD, APX, CAT, GR, POX and metabolites – proline, glutathione, ascorbate, β-carotene) and indices of oxidative stress parameters (viz*.* PPO, H_2_O_2_, and MDA).

**Results:**

The results delineated that Cr uptake and indices of oxidative stress were increased with increasing concentration of Cr stress in all the varieties (HJ 541, HJ513 & SSG 59–3) at both the growth stages (35 & 95 DAS). At higher concentration (4 ppm), Cr stress decreased the grain yield (45–50%) as compared with controls. Polyphenoloxidase activity, MDA and H_2_O_2_ content increased at both growth stages in all the varieties. However, antioxidative enzymes and metabolite activities increased due to Cr stress but this increase was not sufficient to counteract with ROS generated under Cr stress which was enhanced on the application of AMF and GB either individually or in combination (spiked in soil). It decreased the indices of oxidative stress and ameliorated the Cr toxicity and increased grain yield (65–70%) in all the varieties.

**Conclusions:**

Both GB and AMF improved the antioxidative activities and stress tolerance capacity of the plant. Glycine betaine at both 50 and 100 mM level, significantly ameliorated Cr toxicity. However, AMF concomitantly with GB further boosts up the amelioration behaviour of the plant against Cr toxicity, at both growth stages in all the varieties. The combination of 100 mM GB with 10 g AMF was observed most effective among all the treatments. Among the varieties, SSG 59–3 had the lowest chromium uptake, indices of oxidative stress, and highest antioxidative system’s activity as compared to HJ 513 followed by HJ 541 variety. Thus AMF and GB either individually or in combination may be used to maintain plant yield attributes under Cr toxicity.

## Background

Sorghum crop [*Sorghum bicolor* (L.) Moench] a member of the family *Poaceae*, is grown worldwide and India ranks second in terms of area under sorghum cultivation after America. It is an important Kharif season cereal crop and is consumed after rice, wheat, maize and barley [1]. Sorghum is a C4 plant that is highly efficient in converting solar energy to chemical energy, and also in water use efficiency [2]. It is directly or indirectly utilized for the nourishment of humans or animals [3]. All these features make it essential to conduct present experiments on sorghum to meet the growing food demands, globally.

Now a day’s increased chromium contamination of agricultural soils has become a global concern. The main sources of Cr (VI) are leather and paint industries. Annually, approx. 2000 to 32,000 tons of Cr is released in the environment from Indian tanning industries only [4]. Chromium is generally found linked with oxygen as oxyanions of chromates (CrO_4_^2−^) or dichromates (Cr_2_O_7_^2−^) in organic matter in soil and aquatic environments [5]. It exists in two stable forms *viz*. trivalent Cr (III) and the hexavalent Cr (VI). Both forms of Cr can interchange and coexist in a dynamic balance regulated by oxidation/reduction, precipitation/dissolution and adsorption/desorption. Both forms may cause toxicity to plants, but Cr (VI) is considered the most toxic form of Cr due to its high solubility and more unstable nature [6]. The allowable dose of Cr in water is set as 8 µg L^−1^ for Cr (III) and 1 µg L^−1^ for Cr (VI). These data suggested providing hexavalent Cr stress in the range up to 4 ppm in the present experiment.

Chromium (VI) acts as a strong oxidant possessing higher redox potential between 1.33 to 1.38 eV causing rapid reactive oxygen species (ROS) generation and resultant toxic effects in plants [7]. Chromium stress in plants is characterized by the decrease in photosynthesis, nutrient uptake, damaging of roots and finally plant death [8, 9]. Some well-established phytotoxic manifestations include replacement of enzyme cofactors and transcription factors, inhibition of antioxidative enzymes, cellular redox imbalance, ionic transport imbalance, DNA damage and protein oxidation [10, 11].

Plants possess several antioxidant defence systems to protect their cells against these stresses and one such system is an accumulation of a variety of small organic metabolites that are collectively referred to as compatible solutes [12]. They act as an osmoprotectant and protects the plant cells from osmotic damages caused by ROS. Compatible solutes include sugars, polyols, glycine betaine (GB), amino acids (proline, histidine) and related compounds [13]. It has been reported that the level of GB increased in plants subjected to abiotic stresses [14, 15]. It functions as an osmoprotectant that suppresses the production of ROS. It counteracts the oxidative stress in plants by elevating the level of proline and antioxidant enzymes like catalase (CAT), Peroxidase (POX) and Superoxide dismutase (SOD). It is a very effective osmoregulating substance [16].

Glycine betaine is a quaternary ammonium compound that is found in plants and mammals etc. [17]. Its level varies considerably among plant species. Many plant species, do not accumulate GB, either in normal or under stressful conditions. In some plants, the natural accumulation of GB is not enough to protect them from abiotic stresses. Under such conditions, exogenous application of GB may help to reduce the adverse effects of various environmental stresses [18]. It is environmentally safe, non-toxic, and water-soluble [19] and there is strong evidence that GB plays an important role in plants to fight against abiotic stresses [20]. Furthermore, *Arbuscular mycorrhizal fungi* (AMF) are recognized as biological agents that potentially increase the tolerance of plants to heavy metal toxicity [21]. Karagiannidis and Hadjisavva-Zinoviadi [22] showed that AMF can enhance yield by simultaneously reducing the chromium content in crop plants. However, no one has reported the amelioration of hexavalent Cr toxicity by using a combined dose of GB and AMF in sorghum.

Therefore GB and AMF were selected to use as an ameliorating agent during the present experiment and the concentrations (50 and 100 mM) were decided based on previous studies conducted on amelioration of heavy metal toxicity using GB in plants [23]. In this research, we tested the hypothesis that whether the combination of GB and AMF ameliorates Cr toxic effects and improves the yield in sorghum. The observations for the amelioration of Cr toxicity were made in terms of rising in antioxidative defence system parameters and fall in oxidative stress parameters. Outcomes of our research would possibly depict a potential way to prevent Cr toxicity in sorghum.

## Results

The present investigation was carried out on three varieties of sorghum *viz*. HJ 541 (single-cut), HJ 513 and SSG 59–3 (multi-cut) to check out the effects of GB (50 & 100 mM), individually and in combination with AMF (10 g) on Cr (VI) toxicity (2 & 4 ppm) given through soil spiking at the time of sowing. The data were collected at 35 and 95 DAS, except for grains weight which was analysed at maturity. The observations were recorded for chromium accumulation; antioxidant defence system enzymes viz. SOD, APX, CAT, GR, POX and metabolites *viz.* glutathione, proline, ascorbate, β-carotene; indices of oxidative stress parameters *viz*. H_2_O_2_, MDA, PPO; and grain yield. The effects of GB and AMF on different physiological and biochemical parameters during Cr stress were studied and analysed.

### Changes in Cr content of different parts of the sorghum plants

**Fig. 1:**
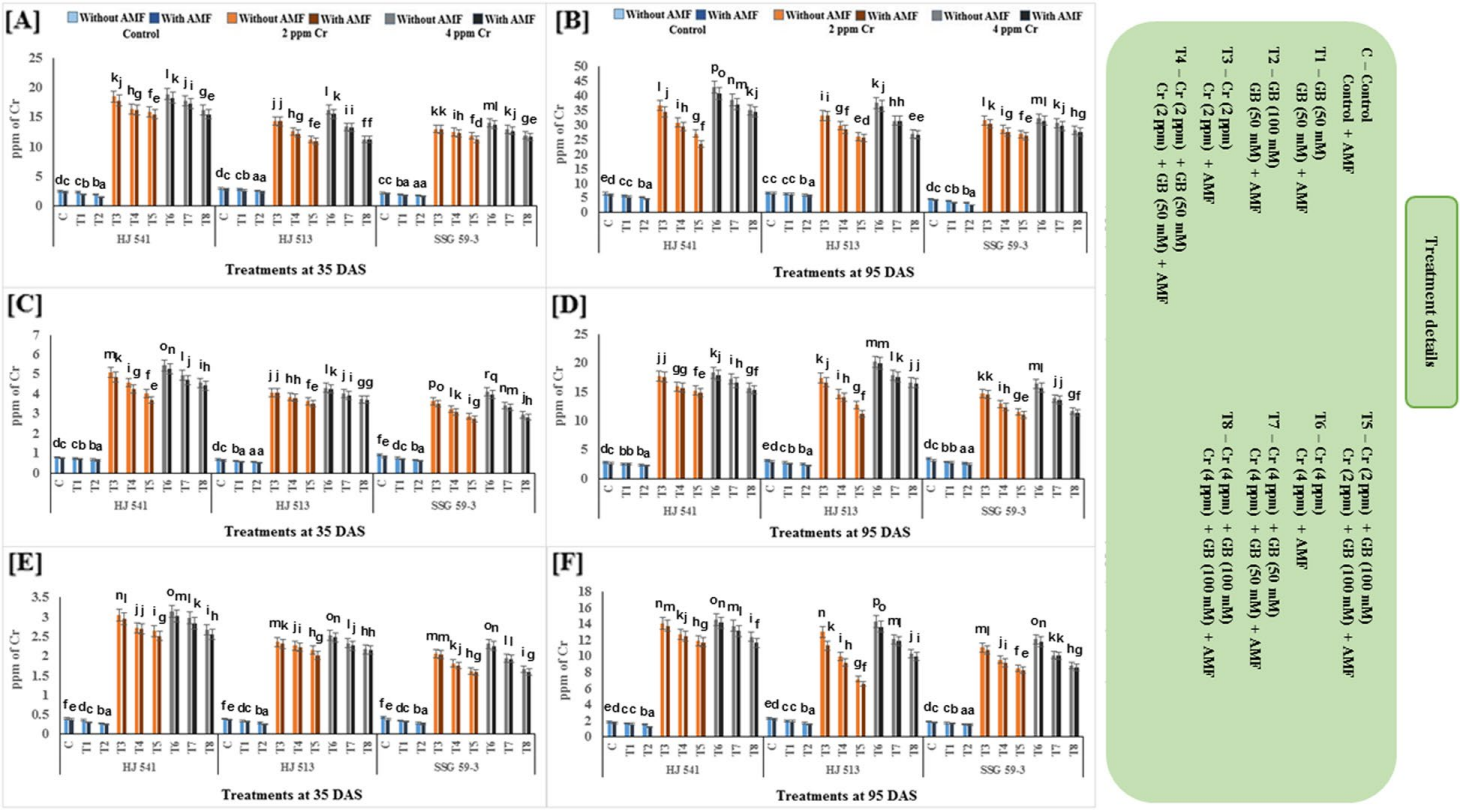
Effects of GB and AMF treatments on Cr accumulation in roots (**A** and **B**), stems (**C** and **D**) and leaves (**E** and **F**) of different varieties of sorghum under Cr (VI) toxicity at 35 & 95 DAS, respectively. Treatment means with different letters in the same column are significantly different from one another according to post hoc Tukey test at (*p* ≤ 0.05); values represent the Means ± SE; *N* = 3, from three independent experiments

The chromium content of roots, stem and leaves were determined at two different growth stages (35 to 95 DAS) in three varieties (HJ541, HJ 513 & SSG 59–3) of sorghum (Fig. 1). The chromium content of all these parts increased with increasing Cr stress, in all the varieties at both growth stages. Chromium content in these parts increased significantly with plant age (35 to 95 DAS) at both levels (2 & 4 ppm) of Cr (VI) in all the varieties. A maximum increase of Cr content was observed at 4 ppm Cr stress in all parts at both growth stages in all varieties. The highest Cr content was observed in roots at 4 ppm Cr stress, during the 95 DAS stage (37.54 ppm), followed by the stem (18.30 ppm) and leaves (13.67 ppm). However, the exogenous application of GB and AMF, either individually or in combination, reduced Cr content in all plant parts, in all the varieties at both growth stages. A maximum decrease in Cr content of roots, stems and leaves was observed in plants provided with the combination of 100 mM GB and AMF at both the growth stages in all the varieties under both Cr stresses (2 & 4 ppm). At 4 ppm Cr stress, the Cr content of roots, stem and leaves was reduced up to 29.48, 14.39 and 10.09 ppm with 100 mM GB and AMF combined application at 95 DAS growth stage. Among the varieties, HJ 541 variety showed the highest Cr content (42.88, 20.20, 14.59 ppm in roots stem & leaves, respectively) followed by HJ 513 (37.56, 18.29, 14.32 ppm in roots stem & leaves, respectively) and lowest in SSG 59–3 variety (32.18, 16.41, 12.11 ppm in roots stem & leaves, respectively).

### Effect of GB and AMF treatments on the anti-oxidative system in sorghum under chromium toxicity

**Fig. 2:**
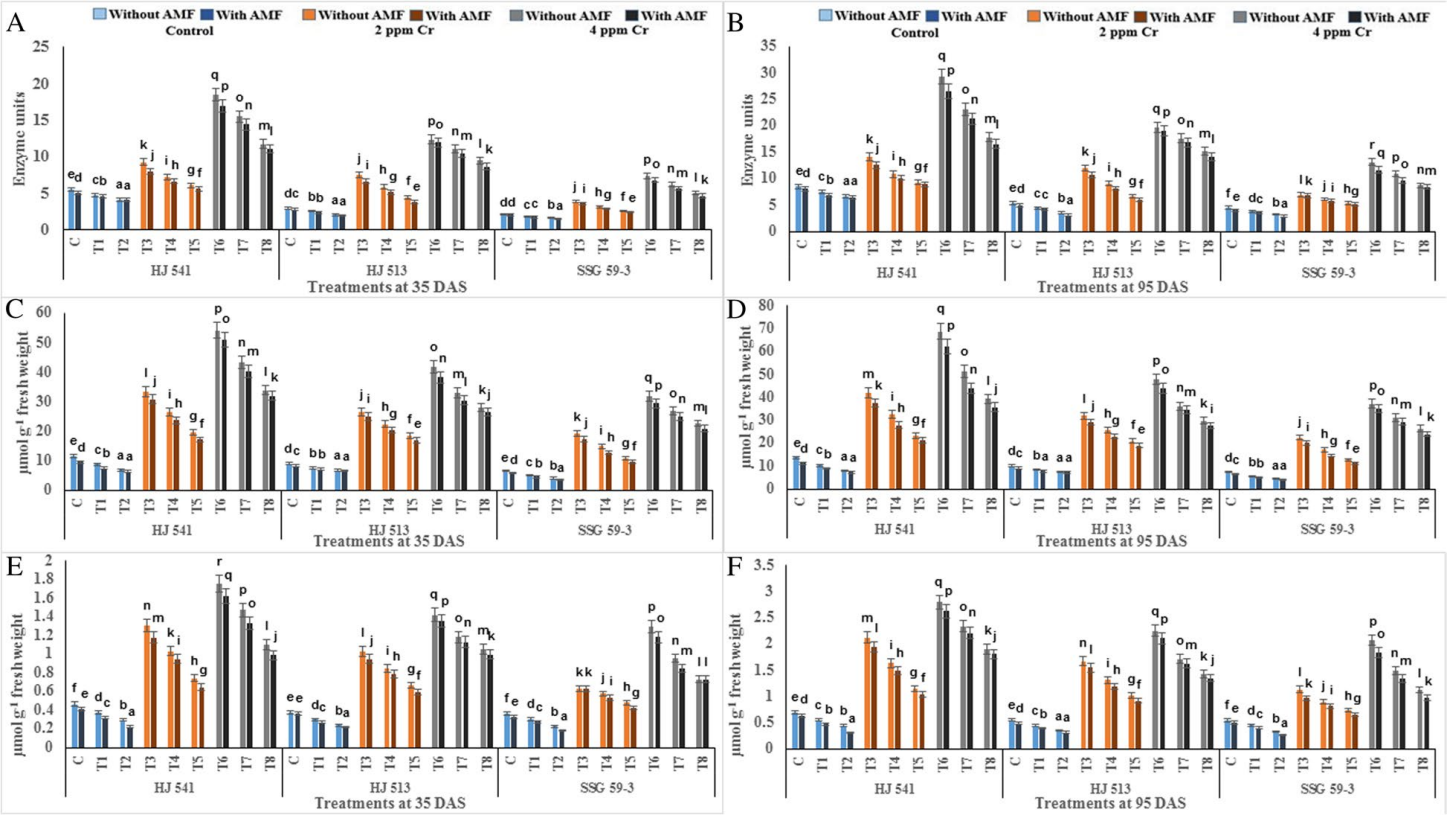
Effects of GB and AMF treatments on indices of oxidative stress viz*.* PPO activity (**A** and **B**), H_2_O_2_ content (**C** and **D**) and MDA level (**E** and **F**) in different varieties of sorghum under Cr (VI) toxicity at 35 & 95 DAS, respectively. Treatment means with different letters in the same column are significantly different from one another according to post hoc Tukey test at (*p* ≤ 0.05); values represent the Means ± SE; *N* = 3, from three independent experiments

The oxidative stress was measured in terms of polyphenol oxidase (PPO) activity, hydrogen peroxide (H_2_O_2_) and malondialdehyde (MDA) contents and antioxidant enzymes and metabolites. PPO causes the oxidation of phenolic compounds and increased oxidative stress. Indices of oxidative stress increased with plant age (35 DAS to 95 DAS) at both levels (2 & 4 ppm) of Cr stress in all the varieties. To observe the effects of GB and AMF on the biochemical qualities of the membrane and oxidative stress in response to Cr stress, PPO activity and contents of H_2_O_2_ and MDA were examined (Fig. 2). An increase in PPO activity, H_2_O_2_ and MDA content was observed under Cr stress as compared with controls at both the growth stages, in all the varieties. However, GB and AMF, individually and their combined application declined PPO activity, H_2_O_2_ and MDA content as compared with Cr alone treatment at both the growth stages in all the varieties. Maximum enhancements in these traits were found when Cr was applied at 4 ppm. At this level PPO activity, H_2_O_2_ and MDA contents were increased by 36.18, 38.12 and 38.92% respectively, without GB and AMF as compared with plants supplied with 100 mM GB combined with AMF at 35 DAS growth stage. Similar trends were observed from the analysis of plants at the 95 DAS growth stage in all the varieties (Fig. 2). Among the varieties, HJ 541 variety showed the highest level of these indices of oxidative stress (29.27 units of PPO, 68.80 and 2.80 µmol of H_2_O_2_ and MDA respectively), followed by HJ 513 (19.73 units of PPO, 47.89 and 2.25 µmol of H_2_O_2_ and MDA respectively) and lowest in SSG 59–3 variety (13.15 units of PPO, 37.16 and 2.08 µmol of H_2_O_2_ and MDA respectively) at 95 DAS. Treatments of GB and AMF decreased indices of oxidative stress, significantly in all the varieties at both growth stages. However, treatments of GB and AMF combined showed the lowest values of these parameters as compared to all other treatments, at both stages in all varieties. Among all the treatments, 100 mM GB with AMF was observed most effective in lowering down the indices of oxidative stress.

**Fig. 3:**
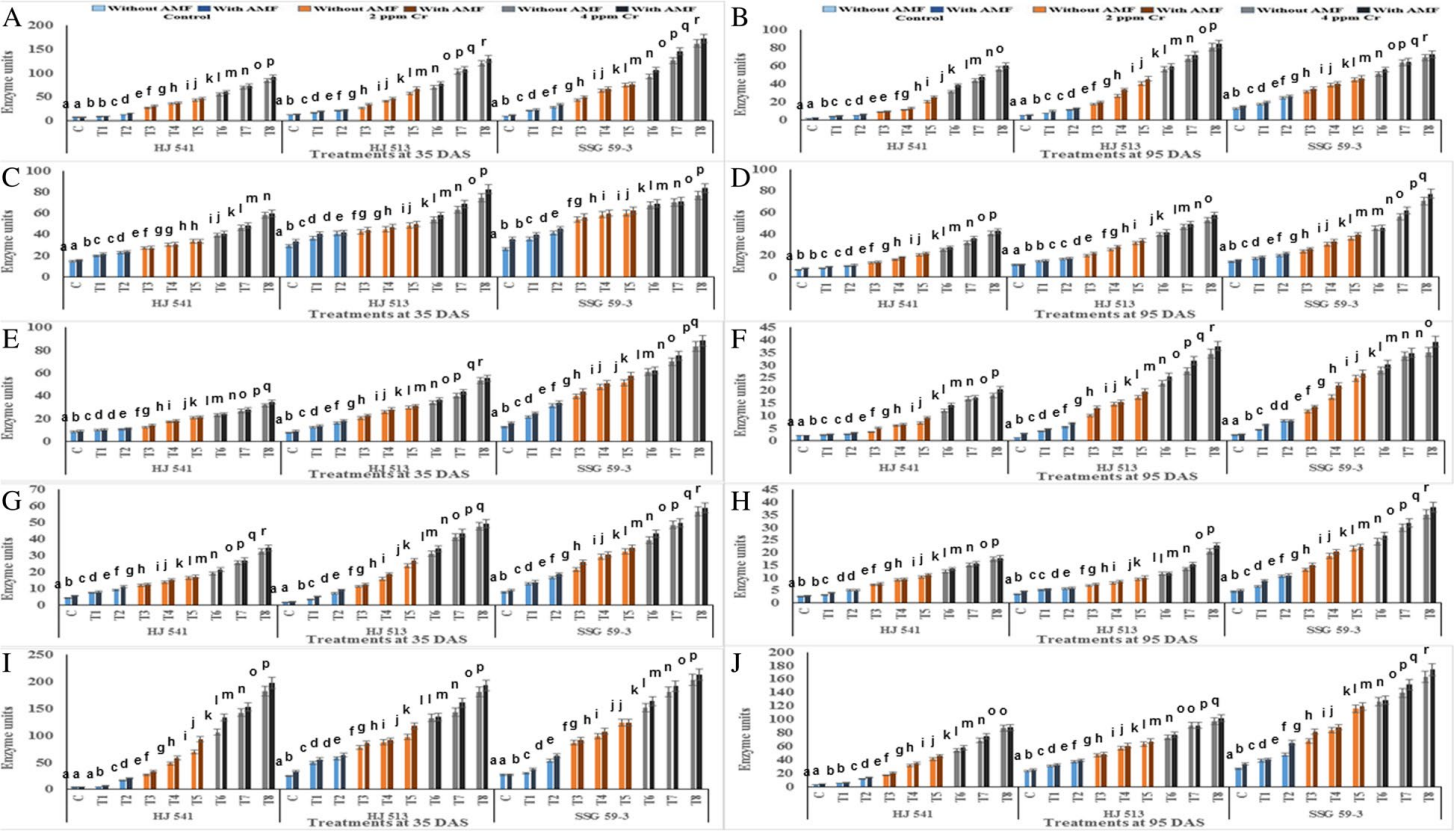
Effects of GB and AMF treatments on activities of antioxidant enzymes viz*.* SOD (**A** and **B**), APX (**C** and **D**), CAT (**E** and **F**), GR (**G** and **H**) and POX (**I** and **J**) in different varieties of sorghum under Cr (VI) toxicity at 35 & 95 DAS, respectively. Treatment means with different letters in the same column are significantly different from one another according to post hoc Tukey test at (*p* ≤ 0.05); values represent the Means ± SE; *N* = 3, from three independent experiments

Furthermore, Cr stress increased antioxidant enzymes SOD, APX, CAT, GR and POX activities in all the varieties at both stages of growth (Fig. 3). Antioxidative enzyme activities increased as the level of Cr stress increased; it was maximum at 4 ppm of Cr stress and tended to increase afterwards because this increase was not enough to protect the plants from oxidative damage caused by the toxicity of hexavalent Cr. The GB and AMF either alone or their combined application further augmented the activities of these enzymes in control as well as in Cr stressed plants in all the varieties at both growth stages. A maximum increase in the activity of these antioxidative enzymes was observed in plants provided with 100 mM GB and AMF in combination, at both the growth stages in all the varieties. However, activities of these enzymes decreased with plant age (35 DAS to 95 DAS) at both levels (2 & 4 ppm) of Cr stress, in all the varieties. At 4 ppm of Cr stress SOD, APX, CAT, GR and POX activities increased up to 87.19, 56.28, 75.38, 85.35 and 85.50% respectively, without GB and AMF, whereas with combination of 100 mM GB and AMF, these increased up to 92.92, 68.99, 83.66, 90.80 and 90.63% respectively, as compared with controls during 35 DAS stage. Similar results were obtained during the 95 DAS stage in all the varieties for enzymatic activities. Among the varieties, SSG 59–3 variety showed the highest activity of these antioxidative enzymes, followed by HJ 513 and lowest in HJ 541 variety (Fig. 3).

The antioxidative defence systems include both enzymatic and non-enzymatic antioxidant components. Apart from enzymatic, non-enzymatic antioxidants such as Glutathione (GSH and GSSG), Ascorbate (AsA), Proline and β-carotene, are crucial for plant defence against oxidative stress. They play a key role as antioxidant buffers. Glutathione reductase is responsible for maintaining the supply of reduced glutathione. It is one of the most abundant reducing thiols in the majority of cells. GSH plays a key role in the cellular control of ROS. The major role of APX is detoxifying hydrogen peroxide in plant cells *via*, ascorbate–glutathione cycle, in which, ascorbate acts as a specific electron donor for APX enzymes in catalyzing the conversion of H_2_O_2_ into H_2_O.

**Fig. 4:**
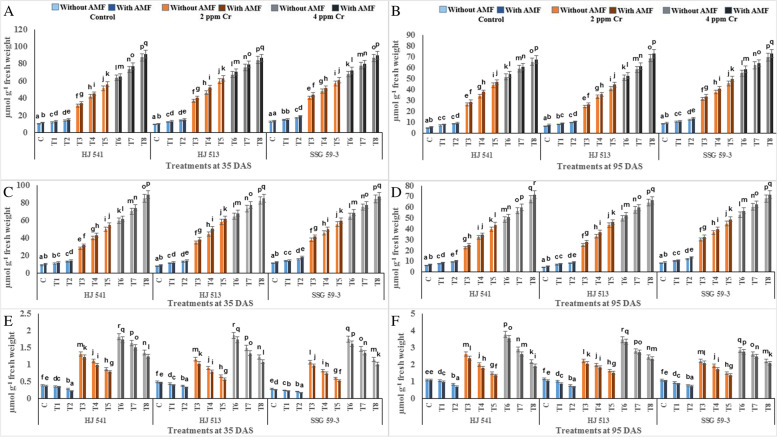
Effects of GB and AMF treatments on antioxidant metabolites *viz.* total glutathione (**A** and **B**), GSH (**C** and **D**) and GSSG (**E** and **F**) in different varieties of sorghum under Cr (VI) toxicity at 35 & 95 DAS, respectively. Treatment means with different letters in the same column are significantly different from one another according to post hoc Tukey test at (*p* ≤ 0.05); values represent the Means ± SE; *N* = 3, from three independent experiments

**Fig. 5:**
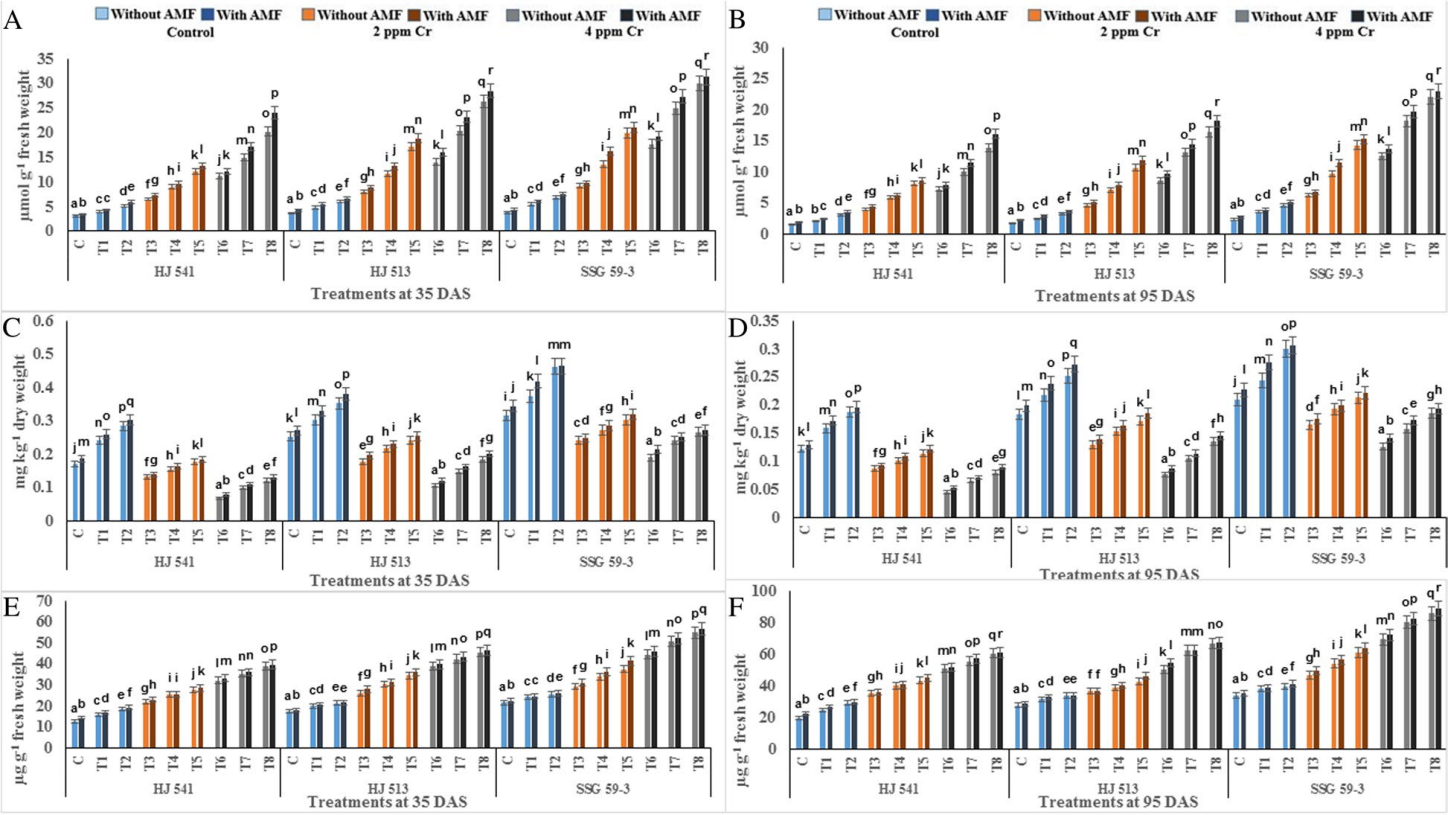
Effects of GB and AMF treatments on antioxidant metabolites *viz.* ascorbate (**A** and **B**), β-carotene (**C** and **D**) and proline (**E** and **F**) in different varieties of sorghum under Cr (VI) toxicity at 35 & 95 DAS, respectively. Treatment means with different letters in the same column are significantly different from one another according to post hoc Tukey test at (*p* ≤ 0.05); values represent the Means ± SE; *N* = 3, from three independent experiments

To determine the ameliorative effect of GB and AMF against hexavalent Cr in sorghum, non-enzymatic antioxidant components were also analysed. Non-enzymatic antioxidant components, namely total glutathione, reduced glutathione (GSH), oxidized glutathione (GSSG), ascorbate, proline and β-carotene were studied. Among them, except β-carotene, all other metabolites increased significantly with increasing concentration of Cr stress at both the growth stages, in all the varieties (Figs. 4 and 5). The β-carotene content decreased significantly with increasing concentration of Cr (VI), at both the growth stages in all the varieties (Fig. 5). All other properties observed were similar to other antioxidative metabolites contents. Along with β-carotene and except GSSG, further increase in the content of these metabolites was observed on exogenous application of GB and AMF, either individually or in combination, at both the growth stages in all the varieties. In contrast, GSSG content decreased on GB and AMF application, either individually or in combination, at both the growth stages in all the varieties (Fig. 4).

Maximum increase in GSH, AsA, proline and β-carotene contents (88.93, 87.50, 64.04, 40% at 35 DAS and 90.99, 90.48, 62.68, 42.85% at 95 DAS respectively), observed in plants provided with the combination of 100 mM GB and AMF, while the content of GSSG decreased maximally (65.76 and 47.64% at 35 and 95 DAS respectively) at same treatment. The level of all these metabolites decreased with plant age at both levels of Cr (VI), but GSSG was increased in all the varieties (Fig. 4). Among varieties, SSG 59–3 variety showed the highest level of GSH, AsA and β-carotene, followed by HJ 513 and lowest in HJ 541 variety while reverse order was observed for GSSG. These findings exemplify the role of GB and AMF in regulating the membrane stability and generation of ROS in cells under the conditions of Cr stress.

### Effect of GB and AMF treatments on grain yield (100 grains weight) in sorghum under chromium toxicity

**Fig. 6:**
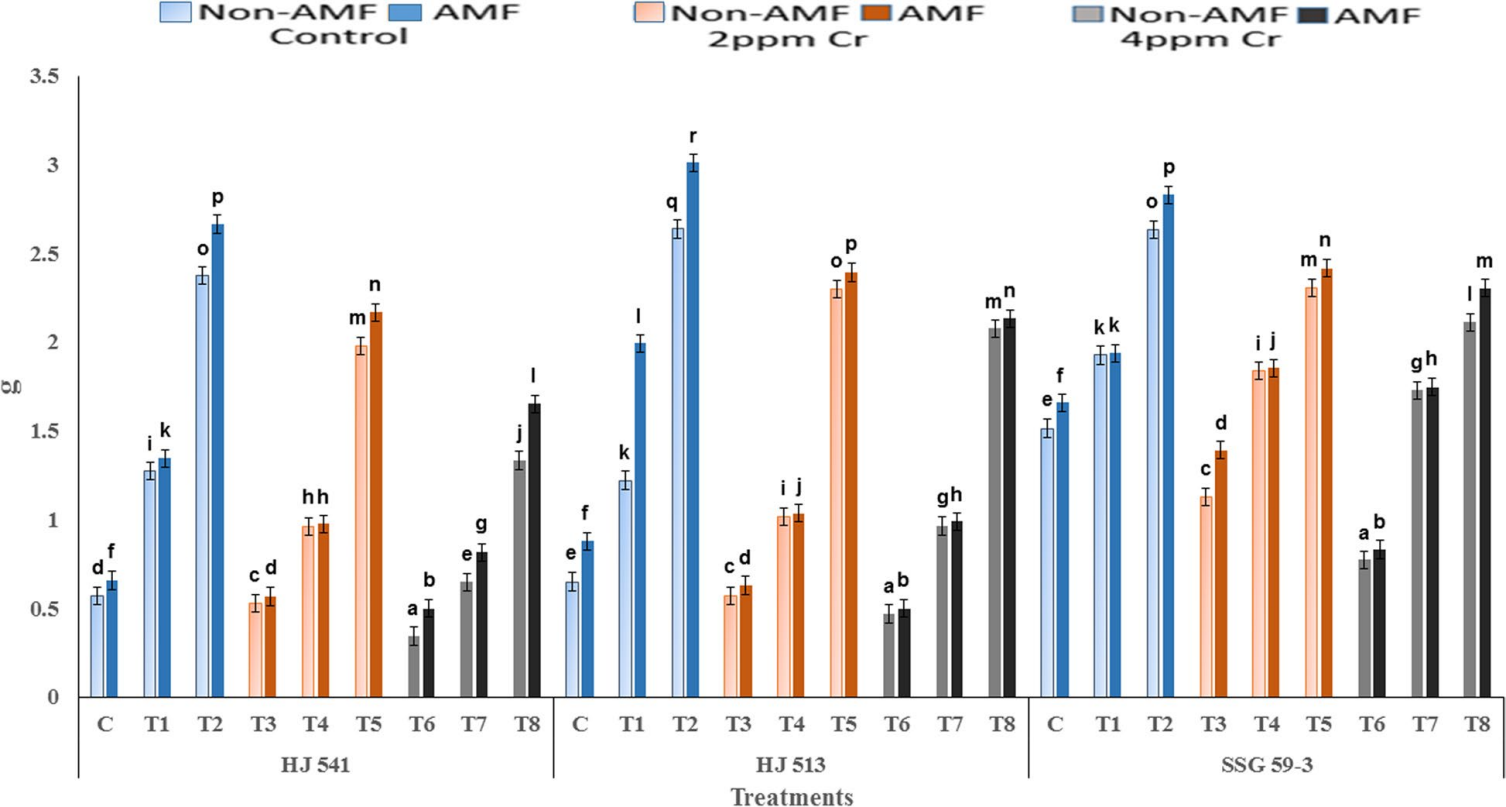
Effects of GB and AMF treatments on grain yield (hundred grains weight in g) in different varieties of sorghum under Cr (VI) toxicity. Treatment means with different letters in the same column are significantly different from one another according to post hoc Tukey test at (*p* ≤ 0.05); values represent the Means ± SE; *N* = 3, from three independent experiments

There was a progressive decrease in grain yield with increasing concentration of Cr (VI), at both the growth stages, in all the varieties (Fig. 6). An increase in grain yield was observed on exogenous application of GB and AMF, either individually or in combination, at both the growth stages in all the varieties. The maximum increase was observed in plants provided with the combination of 100 mM GB and AMF, at both the growth stages in all the varieties.

The results demonstrate that the weight of hundred grains decreased significantly with increasing concentrations of Cr (VI) in all the varieties. However, the application of GB with and without AMF resulted in a significant increase in the weight of hundred grains, in all the varieties. Maximum increase was observed with treatment of 100 mM GB with AMF (2.83, 2.33 & 2.03 g in control, 2 ppm Cr & 4 ppm Cr, respectively), followed by 100 mM GB only (2.55, 2.19 & 1.84 g in control, 2 ppm Cr & 4 ppm Cr, respectively), 50 mM GB along with AMF (1.76, 1.29 & 1.18 g in control, 2 ppm Cr & 4 ppm Cr, respectively), 50 mM GB only (1.47, 1.27 & 1.11 g in control, 2 ppm Cr & 4 ppm Cr, respectively) and AMF only (1.06, 0.86 & 0.61 g in control, 2 ppm Cr & 4 ppm Cr, respectively). The highest grain yield was observed in SSG 59–3 (1.83 g) followed by HJ 513 and HJ 541. The lowest grain yield was observed in HJ 541 (1.19 g).

## Discussion

Chromium toxicity in cultivable lands has become a serious problem all over the world [24]. It reduces the growth and yield of the sorghum crop [25]. There are many reports on Cr (VI) toxicity causing hazardous effects in plants. However, reports on amelioration of chromium toxicity by using GB and AMF together are scanty in literature. In this study, the ameliorative effects of exogenously applied GB and AMF (individually and in combination) against Cr (VI) toxicity on the antioxidative defence system was investigated in sorghum. During present research, increasing levels of Cr treatments resulted in increased Cr content in sorghum. It seems that after the application of only 2 and 4 ppm of Cr, the Cr content in roots, stem and leaves increased many folds i.e. more than the highest treatment of 4 ppm. The reason behind this might be the lower weight of dried sorghum plant as compared to the weight of soil (5 kg pot^−1^) because the concentration of matter changes concerning the weight of medium when it is expressed in terms of weight. It increases as the weight of the medium decreases. Similar reports have been reported earlier also [26, 27]. The Cr content was higher in roots followed by stem and leaves indicated that sorghum plants might have abundant resistance against Cr stress as reported by another researcher in chickpea [28]. Reduction in Cr content of plant samples might be due to GB and AMF, either individually or in combination maintains cell membranes integrity and protects cells from damages which in turn limits the entry of Cr into the cell. The reduction in Cr absorption by plants on GB application might also be due to the shielding nature of GB towards cell membranes that reduces chromium movement to cells [29, 30]. Similar results have been reported for Pb and Cd contents in mung bean [31], rice [32] and wheat [33].

Karagiannidis and Hadjisavva [22] reported that AMF inoculation increased nutrient uptake and suppresses Cr, Mn, Fe, Co, Ni, and Pb absorption in duram wheat. It suggested other possibilities in reduction of Cr absorption with AMF and GB application might be the competition between nutrients and Cr for entry into the cells. Many reports on heavy metal resistant microorganisms have indicated the exceptional ability of AMF to promote the growth of host plant under stressful conditions [24, 34]. Moreover, AMF also has been recognized as a potential biological agent that increases the tolerance capacity of host plant under heavy metal stress.

It was noticed that Cr enhanced ROS generation such as H_2_O_2_ and hydroxyl compounds which in turn increases MDA level and PPO activity. It was reported earlier that Cr is non-essential for plants and generates toxic stress by causing reduction of molecular oxygen and producing intermediate products called ROS such as superoxide radicals, hydroxyl radicals and H_2_O_2_. Interestingly, the generation of ROS is the first line of defence reaction exhibited by any plant cell in response to stress. They further induce the synthesis of other biomolecules (metabolites) and activation of enzymes of various pathways as a defence mechanism. The level of these compounds signifies the extent of stress and are known as indices of oxidative stress. Membrane lipids and proteins are more liable to be attacked by ROS making them reliable indicators of oxidative stress in plants.

In the present study activities of antioxidant enzymes and metabolites were increased with increasing levels of Cr treatments (Figs. 3, 4 and 5). But this increase was not sufficient in scavenging the ROS generated under Cr stress as was evident from increased H_2_O_2_, MDA and PPO activities at the same treatments of Cr. Further, exogenous application of AMF and GB both individually or in combination enhanced antioxidant enzymes and metabolites activities at same Cr treatments in Sorghum and alleviates chromium induced toxicity as was evident from reduced H_2_O_2_, MDA and PPO activities on GB and AMF application (Fig. 2). The reason behind the promotive role of GB and AMF towards antioxidants activities might be the inhibition of Cr absorption and increased nutrient absorption as studied by Jabeen *et al*. [35] in mung bean under Cr toxicity. Moreover, GB itself acts as compatible solutes and AMF helps in the accumulation of them that functions as osmoprotectants and counteracts the oxidative stress by elevating the levels of antioxidant enzymes and metabolites [36]. Hisyam *et al*. [37] have also reported increased antioxidant system activities on exogenous GB application to counteract the stress caused by water deficiency in rice plants. Wang *et al*. [38] were also of a similar view that GB acts as an osmoprotectant, which in turn protects the plant cells from osmotic stresses and resulted in decreased PPO activity while working on GB accumulation in wheat. Raza *et al*. [39] and Gill *et al*. [7] also got similar reports on exogenous GB application in wheat and brassica under Cr toxicity. These reports are supportive of the findings of the present investigation.

In the present experiment loss of grain yield on Cr application was noticed that might be due to excessive production of ROS which is toxic to plants and cause oxidative damage to cellular constituents that resulted in the loss of growth and yield as reported by Khaliq *et al*. [40], who studied the effect of Cd toxicity in duram wheat. The other reason might be increased PPO activity which causes oxidation of polyphenols that reduce the chances of plants growth and reduces the yield under stressful conditions [41, 42]. Apart from that H_2_O_2_ is also a very toxic compound and a higher content of it produces injuries through lipid peroxidation in plant cells which in turn increases MDA content in plants that might also be the cause for reduced yield during stressed conditions [43, 44]. The decrease in grain yield under Cr toxicity may also be due to increased Cr absorption with increasing Cr stress in the plant that caused damaging of roots, chlorosis, necrosis, loss of mineral nutrition, and loss of water balance, ultimately resulted in reduced yield of plants as also suggested by Ali *et al*. [45] in barley, Gill *et al*. [46] in oilseed rape cultivars under Cr toxicity and Kanwal *et al*. [47] in wheat under lead toxicity. The reduction in yield under Cr (VI) toxicity has also been reported widely in literature [23, 47–49].

The results of the present investigation revealed an increased yield on GB and AMF application both individually or in combination (Fig. 6). This increase in grain yield might be resulted due to a reduction in Cr uptake on GB and AMF application which in turn decreased stress level by maintaining proper stomatal conductance, chloroplast ultrastructure, RuBisCo activity, photosynthetic capacity and proper nutrient uptake [50]. Glycine betaine increased the activity of the antioxidant system which in turn prevents plants from oxidative damages caused by ROS generated due to stressed conditions that might result in enhanced grain yield [51–54]. Hassan *et al*. [55] demonstrated similar effects on AMF inoculation in sunflower under Cd toxicity, as were observed during the present investigation. Similarly, GB application increased the growth and yield in rice plants under Cd toxicity [33]. Bharwana *et al*. [56] also obtained similar results that foliar application of GB increased the yield of cotton plants grown under lead (Pb) toxicity. However, the mechanism(s) involved in the enhancement of growth and yield of the plant by GB and AMF application is still not clear. In the present experiment, variety SSG 59–3 showed the highest grain yield as compared to HJ 513 and HJ 541 (Fig. 6). This might be ascribed to the highest level of antioxidant enzymes and metabolites activities (Figs. 3, 4 and 5), and the lowest level of Cr accumulation and indices of oxidative stress parameters (Figs. 1 and 2) in SSG 59–3 variety followed by HJ 513 and lowest in HJ 541.

To sum up, our findings revealed that Cr stresses significantly reduced the grain yield, antioxidant enzymes and metabolites activities. Indices of oxidative stress parameters were dominant due to Cr toxicity. However, the exogenous application of GB and AMF both individually and in combination significantly enhanced the grain yield and reduced the indices of oxidative stress parameters by improving antioxidant enzymes and metabolites activities under Cr stresses. The GB and AMF application also reduced the Cr accumulation and transport. No reports are available about the mechanism of GB and AMF combination in sorghum under Cr stress. Hence, further studies are needed at the field level to see the role of GB and AMF combinations and their mechanism towards various plant species under heavy metal stresses.

## Conclusions

The Cr toxicity (2 & 4 ppm) in sorghum plants resulted in increased ROS levels in all the varieties at both vegetative and grain filling stage. The deleterious effects increased with the increasing concentration of Cr. This may be due to increased Cr uptake which resulted in increased indices of oxidative stress. Though, the components of the antioxidant defence system increased under Cr toxicity but it was not sufficient to combat the toxicity stress. As revealed by a high level of indices of oxidative stress parameters of the plant. Exogenous application of GB and AMF, however, improved the stress tolerance due to further increase in enzymes and metabolites of antioxidant defence system which in turn reduces indices of oxidative stress. The treatment of GB at both 50 and 100 mM level, applied in soil, significantly ameliorated Cr toxicity. However, AMF (10 g) concomitantly with GB, at both 50 & 100 mM level, further ameliorated the effects of Cr toxicity in sorghum plants at both growth stages (35 & 95 DAS). But the AMF application with GB at 100 mM level was found more beneficial at both growth stages and it was observed most effective and best concentration among all the treatments, for the amelioration of Cr toxicity in sorghum plants. However, the antioxidant effects were found more prominent at 35 DAS than 95 DAS. Based on results obtained in the present investigation, variety SSG 59–3 was observed to be more tolerant to Cr toxicity followed by HJ 513 and HJ 541. Further studies in field conditions are necessary to confirm the mechanisms and findings of this experiment.

## Methods

### Plant material selection

The present research was conducted in the screen house of the department of biochemistry, college of basic sciences & humanities, Chaudhary Charan Singh Haryana Agricultural University, Hisar, Haryana (India). Three varieties of sorghum (*Sorghum bicolor* L.) *viz*. HJ-541, HJ 513 and SSG 59–3 were procured from the forage section of the university. These varieties were selected because they are the only source of forage in dryland during the summer season and they are widely grown in the Haryana region. Also, SSG 59–3 is sweeter than HJ 513 (multi-cut) variety and HJ 541 (single-cut) variety. Moreover, HJ 541 is suitable for both grain and fodder yield while HJ 513 is more suitable for grain yield. However, there are no reports about the sensitivity of these three cultivars for GB and AMF, under Cr (VI) toxicity.

### Experimental details and raising of the crop

Three varieties of sorghum at two growth stages *viz*. vegetative (35 DAS) and grain filling (95 DAS) stages were tested for the amelioration of chromium toxicity (2 & 4 ppm) by exogenous application of GB (50 & 100 mM) and AMF in soil both individually and their combination, in completely randomized block design. The seeds of uniform size were selected and surface sterilized with 0.01% mercuric chloride (HgCl_2_) solution for 10 min, followed by 5 times washing with distilled water. The plants were raised in earthen pots lined with polyethene bags filled with 5 kg sandy loam, acid (5% HCL) washed soil. The sterilised seeds were sown at 2 cm depth in the pots. Two weeks old seedlings of the same size were transferred to other pots containing 5 kg soil. Soil properties are mentioned in Table 1. Separate pots were kept for control plants. Three replications were maintained for each treatment and control. All pots were irrigated with equal quantities of water and nutrient solution as per recommended package of practices (POP).

**Table 1:** Physicochemical properties of soil used during the present experiment

**Property**	**Value & unit**	**Evaluation**
Texture	-	Sandy loam
Sand	71.70%	-
Silt	18.96%	-
Clay	9.34%	-
pH	8.2	Basic
OC	0.32	Low
EC	0.17 DS meter^−1^	Normal
Nitrogen (N)	3 mg kg^−1^ soil	Low
Phosphorus (P)	8 mg kg^−1^ soil	Low
Potassium (K)	84 mg kg^−1^ soil	Normal
Zink (Zn)	0.61 mg kg^−1^ soil	Normal
Iron (Fe)	0.7 mg kg^−1^ soil	Low
Copper (Cu)	0.18 mg kg^−1^ soil	Normal
Manganese (Mn)	2.73 mg kg^−1^ soil	Normal
Chromium (Cr)	0.016 mg kg^−1^ soil	Low

### Chemicals and reagents

The chemicals and reagents used during this research work were of high analytical grade. All the chemicals were procured from Sigma Chemicals Co. USA, Sisco Research Laboratories (SRL), Hi-Media and E. Merck Ltd.

### Treatments and growth conditions

During the present research, the treatments were provided based on procedures followed in previous experiments [7]. The detailed composition of treatments used in this experiment is given in Table 2.

**Table 2:** Treatments details of AMF and GB provided in the soil before plantation

**Treatment Name**	**Treatment Composition**	
C	Control	Control + AMF
T1	GB (50 mM)	GB (50 mM) + AMF
T2	GB (100 mM)	GB (100 mM) + AMF
T3	Cr (2 ppm)	Cr (2 ppm) + AMF
T4	Cr (2 ppm) + GB (50 mM)	Cr (2 ppm) + GB (50 mM) + AMF
T5	Cr (2 ppm) + GB (100 mM)	Cr (2 ppm) + GB (100 mM) + AMF
T6	Cr (4 ppm)	Cr (4 ppm) + AMF
T7	Cr (4 ppm) + GB (50 mM)	Cr (4 ppm) + GB (50 mM) + AMF
T8	Cr (4 ppm) + GB (100 mM)	Cr (4 ppm) + GB (100 mM) + AMF

#### Chromium stress treatments

Potassium dichromate salt (K_2_Cr_2_O_7_.7H_2_O) procured from Sigma Ltd. company, was used with distilled water to make two different levels of Cr stress solution (2 and 4 ppm). The soil in each pot was treated with 1 L of respective, out of these two different levels of Cr stress solutions just after plantation of a seedling. The level of respective stress was maintained by supplying respective Cr solution in the respective pots within the 7 days interval.

#### Glycine betaine treatments

Exogenously GB (50 and 100 mM) stalk solutions were prepared with distilled water and 1 L of this from each was supplied in the soil of respective pots just after plantation of a seedling. The level of respective concentration of GB was maintained by supplying respective GB solution in the respective pots within a week interval.

#### *Arbuscular mycorrhizal fungi* (AMF) treatment

The AMF was supplied exogenously in the soil before the plantation of a seedling. The treatment of AMF was provided by mixing 10 g of medium containing AMF in soil per pot. Generally, AMF can grow itself in the moist medium of soil and may increase its levels as time passes. So it was applied only once at the time of plantation of seedling in pots.

### Plant sampling and analysis

The plant samples from control and each treatment were collected at 35 and 95 DAS. A complete plant was collected in an ice-cooled thermal box. It was further divided into leaf, shoot and root. Fresh leaves were used for the estimation of antioxidative enzymes, metabolites and indices of oxidative stress parameters. Shoot samples were hand homogenised and used immediately for the estimation of enzymes activity. Leaf, stem and root samples were dried in an oven for 72 h at 70 °C then Cr contents were estimated separately. The data was analysed by using a three-factorial, analysis of variance ANOVA, CRBD design in SPSS software. Significant (*p* ≤ 0.05) differences between treatments were determined using critical difference.

### Determination of soil properties

The soil was analysed for texture, pH, electrical conductivity, organic carbon, N, P, K, Fe, Mn, Cu, Zn and Cr (Table 2). The texture was determined by the International Pipette method [57]. The pH of the soils was measured with a glass electrode using soil suspension of 1:2 (soil: water) and electrical conductivity in the supernatant as given in [58]. Organic carbon was determined by the wet-oxidation method of Walkley and Black [59]. Available nitrogen (N) was determined by alkaline permanganate method [60], available P content was determined by extracting the soil samples using 0.5 M NaHCO_3_ and analysed by spectrophotometer [61] and available potassium was extracted by using neutral normal ammonium acetate and the content was determined by aspirating the extract into flame photometer [58]. The available forms of Fe, Mn, Cu, Zn and Cr were extracted by DTPA at pH 7.3 and determined using an atomic absorption spectrometer [62].

### Determination of chromium contents

Chromium content was estimated in plant tissue (leaf, stem and roots) sample by using the atomic absorption spectroscopy technique [62]. Five hundred mg tissue sample along with 20 ml digestion mixture (nitric acid and perchloric acid in 4:1 ratio, respectively) was digested overnight in a 100 ml conical flask at room temperature, followed by heating on an electric heater until a very small amount and colourless mixture (2–3 ml) was left in the flask. After cooling the total volume was made up to 25 ml with distilled water. The chromium content was determined in this digested mixture by calibration of standards of Cr (VI) in the form of potassium dichromate in the range 0 – 6 mg L^−1^ in water, and comparing with samples through atomic absorption spectroscopy (AAS). The results were expressed in ppm.

### Determination of the enzymatic antioxidants

Following enzymatic antioxidants, parameters were studied at the vegetative and grain filling stage in sorghum plants.

#### Extract preparation for the estimation of enzymatic antioxidants

The complete extraction procedure was carried out below 4^0^C. Two g of fresh and cleaned leaf tissue was homogenised in 10 ml of 0.1 M potassium phosphate buffer (pH-7.0) by using a previously chilled mortar and pestle. The homogenate was centrifuged at 10,000 rpm for 15 min. The supernatant was collected as crude extract and stored in a refrigerator for total soluble protein estimation. It was used for enzyme assay at the same time.

#### Superoxide dismutase (SOD)

Superoxide dismutase was assayed by measuring its ability to inhibit the photochemical reduction of nitro-blue tetrazolium (NBT) following the method of Beauchamp and Fridovich [63]. The 3.0 ml reaction mixture contained 2.5 ml of 60 mM Tris–HCl (pH 7.8), 0.1 ml each of 420 mM L-methionine, 1.80 mM NBT, 90 µM riboflavin, 3.0 mM EDTA and enzyme extract. Riboflavin was added at the end. The tubes were shaken properly and placed 30 cm below the light source consisting of three 20 W-fluorescent lamps (Phillips, India). The reaction was started by switching on the light and terminated after 40 min of incubation by switching off the light. After terminating the reaction, the tubes were covered with black cloth to protect them from light. A non-irradiated reaction mixture was kept that did not develop any colour and served as control. A separate blank was prepared for each sample, simultaneously by taking boiled enzyme extract. The reaction mixture without enzyme extract had developed maximum colour and its absorbance was decreased with the addition of enzyme. The amount of inhibition was used to quantify the enzyme. The absorbance was recorded at 560 nm. The volume of enzyme extract used in 50% inhibition of the photochemical reaction was considered as one enzyme unit. One enzyme unit was the amount of enzyme required to inhibit the photo-reduction of one µmole of NBT. The enzyme activity was expressed in terms of unit g^−1^ fresh weight and was converted to unit mg^−1^ protein by estimating the total soluble proteins in the sample. The per cent inhibition was calculated by following a formula of Asada *et al*. [64].

$$Percent inhibition=\frac{V-v}{v}\times 100$$

where.

V = Rate of assay reaction in absence of SOD.

v = Rate of assay reaction in presence of SOD.

#### Ascorbate peroxidase activity (APX)

Ascorbate peroxidase was assayed by the method of Nakano and Asada [65]. Three ml reaction mixture contained 2.7 ml of 100 mM potassium phosphate buffer (pH 7.0), 0.1 ml L-ascorbate and 0.15 ml H_2_O_2_. The reaction was initiated by adding 50 µl of enzyme extract. A decrease in absorbance was recorded at 290 nm spectrophotometrically for 2 min against a suitable blank. A separate blank was prepared for each sample, simultaneously by taking boiled enzyme extract. The enzyme activity was calculated, using the molar extinction coefficient (Absorbance of one molar solution) of 2.8 mM^−1^ cm^−1^ for ascorbate in the standard equation for absorbance. One enzyme unit corresponds to the amount of enzyme required to oxidize one nmol of ascorbic acid min^−1^.

The standard equation for absorbance as *A* = *ε* × *Ɩ* × *c.*

Where *A* is the amount of light absorbed by the sample at a given wavelength, *ε* is the molar extinction coefficient, *Ɩ* is the distance that the light travels through the solution, and *c* is the concentration of the absorbing species.

#### Catalase activity (CAT)

The activity of the enzyme was measured by a slightly modified method of Sinha [66]. The reaction mixture contained 0.55 ml of 0.1 M potassium phosphate buffer (pH 7.0), 0.4 ml of 0.2 M hydrogen peroxide and 50 µl of enzyme extract. It was mixed thoroughly and incubated for 1 min at room temperature followed by the addition of 3.0 ml dichromate reagent to it. A separate reaction was run for control, comprising 0.6 ml potassium phosphate buffer and 0.4 ml hydrogen peroxide (0.2 M), without enzyme extract. The tubes were kept in a boiling water bath for 10 min. After cooling, the absorbance was recorded at 570 nm using a suitable blank containing boiled enzyme extract. The absorbance of the sample was subtracted from that of control and the amount of hydrogen peroxide was calculated from a standard curve. One enzyme unit corresponds to the amount of enzyme required to break down one µmole of hydrogen peroxide min^−1^ or mg^−1^ protein.

#### Glutathione reductase activity (GR)

Glutathione reductase was assayed using the procedure of Halliwell and Foyer [67]. The assay mixture (3.0 ml) contained 2.5 ml of assay buffer, 0.2 ml EDTA, 0.15 ml of 50 mM oxidized glutathione, 0.1 ml of 30 mM NADPH and 50 µl of enzyme extract. Assay reaction was initiated by adding NADPH at the end. A decrease in absorbance was recorded simultaneously, at 340 nm wavelength against a suitable blank containing boiled enzyme extract. The amount of NADPH oxidized was calculated by using an extinction coefficient (Absorbance of one molar solution) of 6.12 mM^−1^ cm^−1^ in the standard equation for absorbance. One unit activity of the enzyme corresponded to the amount of enzyme required in the oxidation of one nmol of NADPH min^−1^.

The standard equation for absorbance as *A* = *ε* × *Ɩ* × *c.*

Where *A* is the amount of light absorbed by the sample at a given wavelength, *ε* is the molar extinction coefficient, *Ɩ* is the distance that the light travels through the solution, and *c* is the concentration of the absorbing species.

#### Peroxidase activity (POX)

Peroxidase was assayed by the method of Shannon *et al*. [68]. The enzyme was assayed by putting 3.5 ml of assay buffer, 0.3 ml of o-dianisidine and 0.1 ml of diluted enzyme extract, in a cuvette of 5 ml capacity. The solution was mixed well. The assay reaction was initiated by adding 0.1 ml of 0.2% hydrogen peroxide followed by recording the change in absorbance at 430 nm wavelength, simultaneously. A separate blank was prepared for each sample, simultaneously by taking boiled enzyme extract. The enzyme activity was expressed as a change in 0.01 absorbance min^−1^ mg^−1^ protein.

#### Polyphenol oxidase (PPO)

Polyphenol oxidase enzyme activity was assayed by the method of Taneja and Sachar [69]. The assay mixture contained 1.8 ml of assay buffer, 2 ml catechol solution as substrate and 0.2 ml enzyme extract in glass test tubes. These test tubes were incubated at 37 °C for 1 h to take place the assay reaction followed by measuring absorbance at 430 nm on a UV–Vis spectrophotometer. A separate blank was prepared for each sample, simultaneously by taking boiled enzyme extract. The enzyme activity was expressed as a change in 0.01 absorbance min^−1^ mg^−1^ protein.

### Determination of the antioxidant metabolites

Following antioxidative metabolites were studied at vegetative and grain filling stage in sorghum plants under different treatments.

#### Glutathione

The level of oxidized, reduced and total glutathione was estimated by the method of Smith [70].

#### Extract preparation

One g of fresh leaf tissue was homogenised in 10 ml of 5% (w/v) sulphosalicylic acid using glass beads as abrasive, at 4ºC. Then, it was centrifuged at 30,000 × g for 20 min (4ºC) and the supernatant was collected for glutathione determination.

#### Assay

Total glutathione (GSH + GSSG), was determined by adding 0.1 ml of 0.5 M potassium phosphate buffer (pH 7.5), 0.5 ml of 0.1 M sodium phosphate buffer (pH7.5) containing 5 mM EDTA, 0.1 ml of 2 mM NADPH, 0.1 ml of glutathione reductase, 0.15 ml of 0.6 mM DTNB and 0.05 ml supernatant in a cuvette. The content was mixed thoroughly before the addition of supernatant, and the reaction was initiated by adding supernatant at the end of the addition process. A separate blank tube was prepared by avoiding the addition of supernatant. The reduction rate of DTNB was monitored at 412 nm for 3 min. Total glutathione content was calculated from a standard curve of GSH (200–400 ng) plotted against the rate of increase of absorbance at 412 nm. Further, the oxidised glutathione (GSSG) content was determined by adding 1.5 ml potassium phosphate buffer (0.5 M, pH 7.5) and 0.2 ml 4-vinyl pyridine to 1 ml supernatant in a test tube. The mixture was allowed to react for 1 h to remove reduced glutathione (GSH). The GSSG content was measured using the same procedure as for total glutathione determination but with a GSSG standard curve (50–200 ng). Reduced glutathione (GSH) content was calculated by subtracting GSSG from the total glutathione content.

#### Proline

The proline content was estimated by the method of Bates *et al*. [71].

#### Extract preparation

One g of fresh leaves sample were homogenised in 10 ml of 3% sulphosalicylic acid and centrifuged at 3000 rpm for 10 min. The supernatant was collected and used for proline estimation.

#### Assay

The extract was filtered through Whatman No. 2 filter paper. Two mL of filtrate along with 2 mL of glacial acetic acid and 2 mL acid ninhydrin were transferred in a test tube followed by heating in the boiling water bath for 1 h. The reaction was terminated by placing the tube in an ice bath. Four mL toluene was added to the reaction mixture and stirred well for 20–30 s. The Toluene layer was separated and cooled to room temperature. The red coloured intensity of toluene was measure at 520 nm. The amount of proline present in the samples was determined from the standard curve (0.04 – 0.2 µg ml^−1^) of proline.

$$Proline content \left(\mu moles per g tissue\right)= \frac{\mu g proline per ml\times ml toluene\times 5}{115.5\times g sample}$$

where 115.5 is the molecular weight of proline.

#### Ascorbic acid

Ascorbic acid was determined by the slightly modified procedure of Oser [72].

#### Extract preparation

One g of the plant tissue was homogenised in 6 ml of ice-cold 0.8 N HClO_4_ and centrifuged at 4^0^C, 10,000 rpm for 30 min. The supernatant was collected and neutralized with 5 M K_2_CO_3_. It was centrifuged again at the same conditions (4^0^C temperature, 10,000 rpm for 30 min). Thus a clear supernatant was obtained, which were used for estimation of ascorbic acid content.

#### Assay

For estimation of total ascorbate, 1 ml extract was treated with equal volume (i.e. 1 ml) of 10% TCA. It was incubated in ice for 5 min. It was further mixed with 1 ml each of 5 M NaOH, 10 mM dithiothreitol (DTT) and 0.5% (w/v) N-ethyl maleimide (NEM) and 2 ml sodium phosphate buffer (pH7.4) in a final volume of 7 ml followed by 1 ml of 2% dinitrophenyl hydrazine and a drop of 10% thiourea, addition. Then the tubes were shaken vigorously and kept in a boiling water bath for 15 min and cooled. After cooling 80% H_2_SO_4_ was added to the tubes at 4 °C and vortexed. Then the absorbance was recorded at 530 nm against a suitable blank without the sample extract. The amount of ascorbate was determined by using a reference curve (0–100 nmols) of ascorbate and expressed as µmoles g^−1^ fresh weight.

#### β-Carotene

The amount of β-carotene was determined by the method of AOAC [73].

#### Assay

A homogeneous suspension was made by dispersing 10 g of shoot sample in 50 ml of water-saturated n-butanol (The n-butanol and water were mixed in the ratio of 6:2 (v/v) and shacked vigorously. Then it was allowed to stand, till it separates into two phases. The upper clear layer was water-saturated n-butanol). After vigorous shaking, it was allowed to stand overnight (16 h) at room temperature in dark. It was shacked again followed by filtration through Whatman filter paper No. 1. The total volume of the filtrate was made up to 100 ml. The absorbance (A) of the clear filtrate was measured at 440 nm in Spectronic-20/spectrophotometer against a blank of saturated n-butanol. The amount of β-carotene was calculated from the following equation:

$$\beta -carotene content \left(ppm\right)=0.0105+23.5366\times A$$

#### Detection of indices of oxidative stress

Following metabolites were studied as indices of oxidative stress at the vegetative and grain filling stage in different treatments during the experimental analysis.

#### Hydrogen peroxide (H_2_O_2_)

##### Extraction

Two g tissue was macerated in 5 ml of ice-cold 0.01 M phosphate buffer (pH 7.0) and centrifuged at 8000 × g for 10 min. The supernatant was collected and used for the estimation of H_2_O_2_ content [74].

##### Assay

Fifty µl of extract were added to 1.95 ml of 0.01 M potassium phosphate buffer (pH 7.0) and 2 ml of dichromate reagent to the mixture. It was kept in a boiling water bath for 10 min and then cooled. After cooling, the absorbance was taken at 570 nm wavelength against a reagent blank without sample extract and the quantity of H_2_O_2_ was calculated from the standard calibration curve (10 to 160 µmole of H_2_O_2_).

#### Malondialdehyde (MDA)

##### Extraction

One g tissue were homogenized in 5 ml of TCA (0.1% trichloroacetic acid; w/v) and centrifuged at 8000 × g for 15 min. The supernatant was used for MDA estimation by the method of Heath and Packer [74].

##### Assay

The MDA estimation reaction was started by putting 1 ml of the supernatant, 4 ml of 20% TCA containing 0.5% 2-thiobarbituric acid (TBA). The content was heated in a boiling water bath at 95ºC for 30 min with constant stirring. Then it was cooled quickly in an ice bath followed by centrifugation at 8000 × g for 10 min. The supernatant was decanted and the absorbance was recorded at 532 nm against distilled water as blank. The values for non-specific absorption at 600 nm were subtracted from it and the concentration of MDA was calculated by using the molar extinction coefficient at 155 mM^−1^ cm^−1^.

### Grain yield determination

The grain yield was determined on 100 grains weight basis. One hundred grains from each replication were selected randomly and weighed, separately for each treatment, by using a laboratory weighing balance. The average value of all replications was calculated and expressed as the yield in grams per 100 grains weight basis.

### Statistical analysis

The present study was carried out in a completely randomized block design (CRBD) with three replications per treatment. All the results were analysed by using IBM SPSS Statistics 23 software for windows [75]. Comparison between different treatments was evaluated with a post hoc test followed by Tukey test. In the present study, the value for *p* was ascertained significant at ≤ 0.05.

## Data Availability

All data generated or analysed during this study are included in this published article [and its supplementary information files].

## Abbreviations

DAS: Days after sowing
GB: Glycine betaine
AMF: *Arbuscular mycorrhizal fungi*
SOD: Superoxide dismutase
APX: Ascorbate peroxidase
CAT: Catalase
GR: Glutathione reductase
POX: Peroxidase
PPO: Polyphenol oxidase
H_2_O_2_: Hydrogen peroxide
MDA: Malondialdehyde
Ppm: Parts per million
ROS: Reactive oxygen species
